# The INCH-Trial: a multicentre randomized controlled trial comparing the efficacy of conventional open surgery and laparoscopic surgery for incisional hernia repair

**DOI:** 10.1186/1471-2482-13-18

**Published:** 2013-06-07

**Authors:** Marijn Poelman, Jan Apers, Han van den Brand, Huib Cense, Esther Consten, Jort Deelder, Boudewijn Dwars, Nanette van Geloven, Elly de Lange, Johan Lange, Rogier Simmermacher, Maarten Simons, Eric Sonneveld, Hermien Schreurs, Jaap Bonjer

**Affiliations:** 1 VU University Medical Centre, Amsterdam, Netherlands; 2 Alkmaar Medical Centre, Alkmaar, Netherlands; 3 Leeuwarden Medical Centre, Leeuwarden, Netherlands; 4 Red Cross Hospital, Beverwijk, Netherlands; 5 Meander Medical Centre, Amersfoort, Netherlands; 6 Slotervaart Hospital, Amsterdam, Netherlands; 7 Tergooi Hospital, Hilversum, Netherlands; 8 Erasmus Unversoty Medical Centre, Rotterdam, Netherlands; 9 Utrecht University Medical Centre, Utrecht, Netherlands; 10 OLVG Hospital, Amsterdam, Netherlands; 11 Westfries Gasthuis Hospital, Hoorn, Netherlands

## Abstract

**Background:**

Annually approximately 100.000 patients undergo a laparotomy in the Netherlands. About 15,000 of these patients will develop an incisional hernia. Both open and laparoscopic surgical repair have been proven to be safe. However, the most effective treatment of incisional hernias remains unclear. This study, the ‘INCH-trial’, comparing cost-effectiveness of open and laparoscopic incisional hernia repair, is therefore needed.

**Methods/Design:**

A randomized multi-center clinical trial comparing cost-effectiveness of open and laparoscopic repair of incisional hernias. Patients with a symptomatic incisional hernia, eligible for laparoscopic and open incisional hernia repair. Only surgeons, experienced in both open and laparoscopic incisional hernia repair, will participate in the INCH trial. During incisional hernia repair, a mesh is placed under or on top of the fascia, with a minimal overlap of 5 cm. Primary endpoint is length of hospital stay after an incisional hernia repair. Secondary endpoints are time to full recovery within three months after index surgery, post-operative complications, recurrences, mortality and quality of life.

Our hypothesis is that laparoscopic incisional hernia repair comes with a significant shorter hospital stay compared to open incisional hernia repair. A difference of two days is considered significant. One-hunderd-and-thirty-five patients are enrolled in each treatment arm. The economic evaluation will be performed from a societal perspective. Primary outcomes are costs per patient related to time-to-recovery and quality of life.

The main goal of the trial is to establish whether laparoscopic incisional hernia repair is superior to conventional open incisional hernia repair in terms of cost-effectiveness. This is measured through length of hospital stay and quality of life. Secondary endpoints are re-operation rate due to post-operative complications or recurrences, mortality and quality of life.

**Discussion:**

The difference in time to full recovery between the two treatment strategies is thought to be in favor of laparoscopic incisional hernia repair. Laparoscopic incisional hernia repair is therefore expected to be a more cost-effective approach.

**Trial registration:**

Netherlands Trial register: NTR2808

## Background

Incisional hernias are defects of the fascia of the abdominal wall, covered by skin, which can develop after abdominal surgery. Bulging through the scar is visible and palpable when patients are standing or coughing [1,2]. These hernias occur in at least 15% of patients after open abdominal surgery within ten years after surgery. Incisional hernias may be asymptomatic, but frequently they cause pain and give aesthetic complaints. They can also cause serious complications like strangulation of the bowel. The quality of life in these patients as well as their chances for employment is reduced [2].

Pre-disposing factors to get an incisional hernia are obesity, which is increasing rapidly in the Western world, and a post-operative surgical site infection [3]. There are no differences between men and women in developing an incisional hernia. Ethnical differences are not known. The pathogenesis of incisional hernias is complex; altered collagen metabolism and extra-cellular matrix disorders causing wound-healing disorders have been found in patients who developed incisional hernias [4].

A population based study showed a 3,7% yearly increase in the incidence of incisional hernia repair per 10.000 people [5] in the United States. Since obesity plays an important role in developing an incisional hernia and is an increasing problem in the Netherlands, we expect the incidence of incisional hernias to increase in the Netherlands as well. Mean age at time of the surgical repair is 58 years old, mean SD 15 years [6]. The majority of these patients will have to go back to work.

Eighty percent of the patients with an incisional hernia undergo surgical repair [5]. The morbidity of open incisional hernia repair is more than 20% involving recurrence and mesh infection. Laparoscopic surgery tends to be safe and is associated with less infections and shorter hospitalization. It is highly feasible in obese patients, because of a good exposure of the incisional hernia. However, the surgical procedure can be difficult and the operating time might be longer. Up till now it is not clear what is the best treatment strategy for incisional hernias. The potential benefits of a more defined treatment strategy includes a shorter hospital admission, cost reduction and less post-operative complications.

Two recent meta-analysis [7,8] state that laparoscopic repair is at least as effective and might be superior to the open approach in a number of outcomes. Total hospital stay was shorter and less post-operative complications were seen. The largest study in the meta-analysis [8] has several shortcomings; randomization is not listed and there was no sample size calculation. Most studies only provide short-term follow-up evaluation and cost-effectiveness is not evaluated. This study, comparing the cost-effectiveness of open and laparoscopic incisional hernia repair, is therefore needed.

Criteria to recommend a surgical repair should be stated and the natural course of an incisional hernia should be examined.

This is a multi-centre study with surgeons who are experienced in open as well as laparoscopic surgery. The study group exists of dedicated laparoscopic surgeons, committed to improve hernia-care. First, we want to know if laparoscopic repair is more effective than open repair. The future of this study will allow evidence-based change of practice.

## Methods/Design

A randomized multi-centre trial comparing the cost-effectiveness of two surgical techniques for the repair of incisional hernias: laparoscopic vs. conventional open repair.

### Inclusion criteria

**Figure 1:**
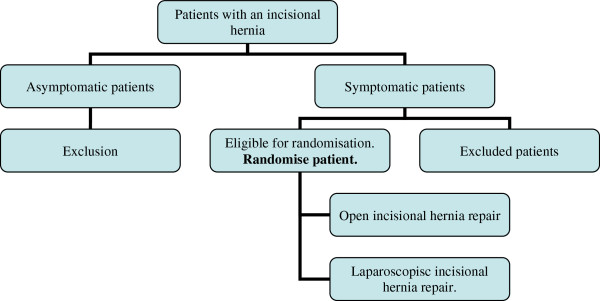
Flowchart.

The following patients will be eligible for the randomization to either open or laparoscopic repair: Adult patients who are referred to the surgical clinic for assessment of an incisional hernia, either primary or recurrent. Imaging of the abdomen will only be done when it is unclear whether an incisional hernia is present. The need for surgery will be determined; pain, severe discomfort and episodes of visceral incarceration are indications for surgery. Only symptomatic patients will get a surgical correction of the incisional hernia. Patients whose incisional hernia is suitable for laparoscopic repair are included in the trial, this decision is at the discretion of the surgeon. After consenting to the study, the patient will be randomized to either open or laparoscopic repair. Patients who are excluded or who don’t want to participate will be registered. (Figure 1; flowchart).

### Exclusion criteria

1. Pregnancy

2. Age less than 18 years old 18

3. Abdominal ostomy

4. History of open abdomen treatment

5. Mentally or cognitively unable to be consented

6. A life expectancy of less than one year

7. Immune-compromised patients

8. ASA > 3 (ASA: scoring system of the American Society of Anaesthesiologists)

### Treatment

Patients will be randomized, using a computer-program, to one of the following surgical approaches:

I) **Open repair** of the incisional hernia: the employed open technique is at the discretion of the participating surgeon. There is no evidence, which open technique, is best; bridging as well as augmenting techniques might be used. Onlay, sublay as well as CST technique are allowed as long as a mesh is used. An overlap of at least 5 cm of the mesh over the fascia is preferable.

II) **Laparoscopic repair** of incisional hernias will entail employment of mesh that is placed subfascially with a minimal overlap of 5 cm. Choice and fixation of the mesh is at the discretion of the surgical team. During laparoscopic surgery a photograph will be taken of the hernia defect before and after the correction is done. When laparoscopic repair is not successfully achieved or complications occur, the surgeon may decide to change to the open surgical procedure (i.e. conversion); this is common practice in laparoscopic surgery.

In each approach, the use of a mesh is preferable, as this has shown to reduce the recurrence-rate [9]. Every detail of the technique should be described. Dutch hernia experts will perform the surgical corrections.

### Case record form

**Figure 2:**
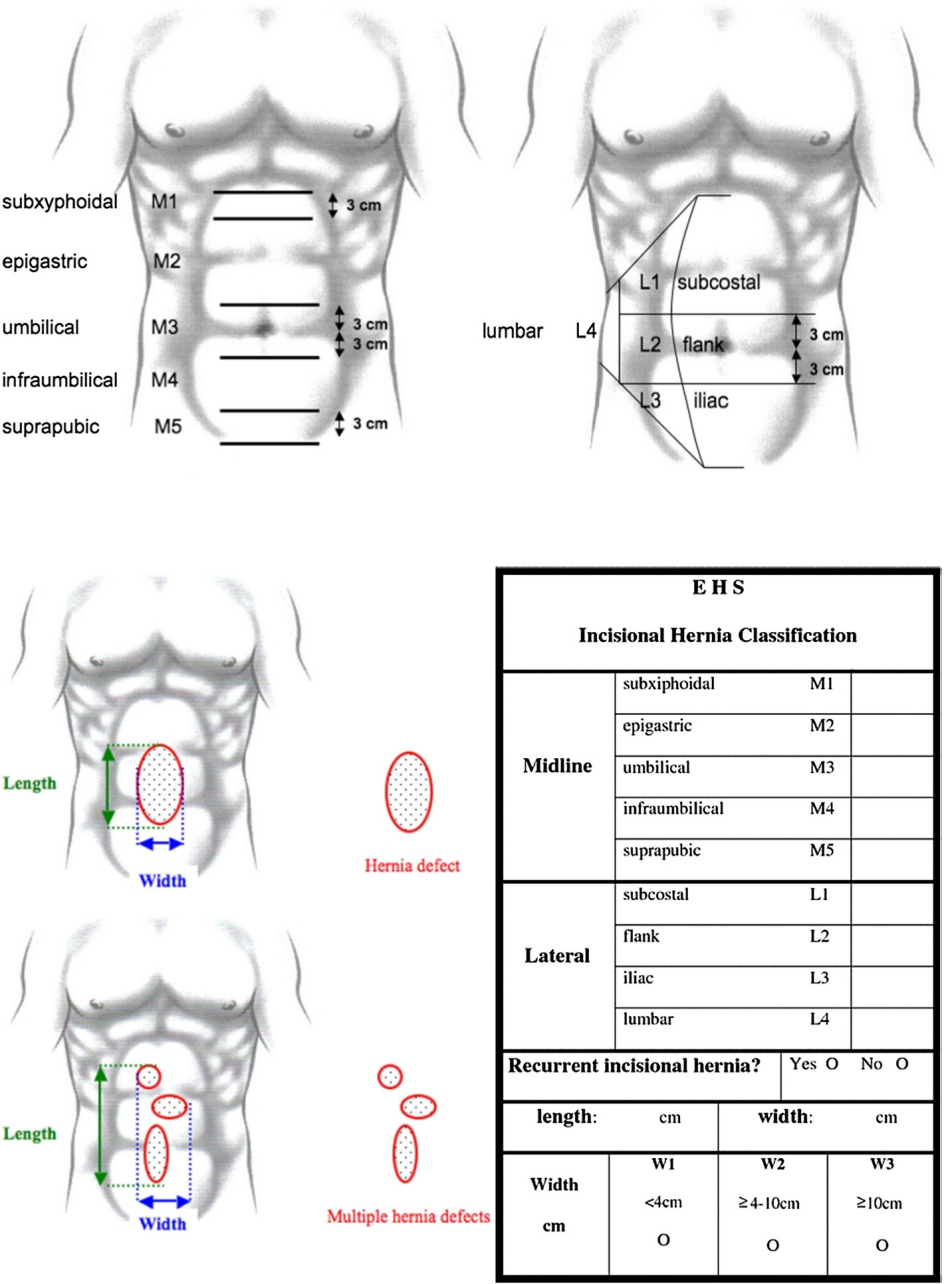
Classification of incisional hernias according tot the European Hernia Society.

*At first presentation* in the outpatient clinics: age/sex/co-morbidity/pre-illness/working-social activities/surgical history/symptoms of the incisional hernia/classification of the incisional hernia:

The European Hernia Society (EHS) tried to categorize incisional hernias, in order to be able to compare different scientific incisional hernia research [10]. A classification system of abdominal wall hernias was formulated (Figure 2). This classification will be used in the INCH-trial.

Postoperatively: open or laparoscopic/length of the scar/duration of surgery/type of mesh used/size of the mesh/type of mesh fixation/presence of bleeding/accidental bowel lesion/use of tubes/use of per-operative antibiotics/possible re-operation.

Inpatient: daily VAS score/use of analgesics/length of hospital stay/morbidity/mortality/QOL at two days post-surgery.

During Follow-up at 2 weeks and 3 months: healthcare and lost productivity costs/QOL at 2 weeks and 3 months post-surgery will be measured through the Short Form 36 and the Carolina Comfort Scale/time to return to work/recurrence/pain/wound infection/patient satisfaction/other morbidity.

During long-term follow-up at 1, 3 and 5 years: Recurrence/pain/wound infection/patient satisfaction/other morbidity.

**Primary outcomes** are:

Length of hospital stay. This is the time until discharge. A patient can be discharged when he/she is able to move normally, tolerate a normal diet and has a VAS painscore < 5 without the use of opiates.

Quality of life measures through SF-36 and CCS.

**Secondary endpoints** are:

Re-operation rate for recurrence or complications of the incisional hernia repair. The analysis will be continued after the cost-effectiveness study has ended, a longer follow-up is needed to examine the recurrence-rate.

28 days post surgery morbidity and mortality,

Total mean costs will be related to the following effect measures in the cost-effectiveness analyses:

1) Time to full recovery.

2) Quality-adjusted life-years (QALYs) based on the SF-36D [11].

Intention to treat analysis will be performed. Blinding is impossible as the surgical difference is visible from the outside.

### Economic evaluation

The aim of the economic evaluation is to describe the costs of laparoscopic and open repair of incisional hernias, and to relate the costs to the clinical effects of the treatments. The time horizon of the economic evaluation is 3 months. A societal perspective is chosen for this economic evaluation. For the measurement and valuation of the costs the Dutch costing guidelines will be used [12].

#### Cost measurement and valuation

Health care utilization will be measured using hospital data and cost diaries during hospitalization and after 2 weeks and 3 months of follow-up. Health care costs include costs of the operation, hospital stay, medical supplies, additional examinations (CT, X-ray, laboratory, etcetera), medication, GP care, emergency visits and ambulatory hospital care. Absenteeism from paid and unpaid work and presenteeism at baseline and after 2 weeks and 3 months of follow-up will also be measured.

For the valuation of health care utilization standard prices published in the Dutch costing guidelines will be used [12]. Medication use will be valued using prices of the Royal Dutch Society for Pharmacy. A detailed cost price calculation will be performed to estimate the costs of laparoscopic and open repair of incisional hernias.

### Analysis of cost-effectiveness

The analysis will be done according to the intention-to-treat principle. Missing cost and effect data will be imputed using multiple imputations according to the MICE algorithm developed by Van Buuren [13]. Costs typically have a highly skewed distribution. Policy makers want to have information on the difference in mean total costs between the two treatment-groups to be able to estimate the total health care budget needed for a specific condition [14]. Therefore, bias-corrected and accelerated bootstrapping with 5000 replications will be used to calculate 95% confidence intervals around the mean difference in total costs between the treatment groups. Incremental cost-effectiveness ratios (ICERs) will be calculated by dividing the difference in mean total costs between the treatment groups by the difference in mean effects between the treatment groups. Bootstrapping will be used to estimate the uncertainty surrounding the ICERs, which will be graphically presented on cost-effectiveness planes. Cost-effectiveness acceptability curves and net monetary benefits will also be calculated. Cost-effectiveness acceptability curves show the probability that collaborative care is cost-effective in comparison with usual care for a range of different ceiling ratios thereby showing decision uncertainty [15].

The baseline data of both treatment groups will be described and 95% confidence intervals will be calculated. Additional as-treated analyses will be done, because patients who were planned to have a laparoscopic repair might have had an open correction and vice versa. Differences in primary and secondary endpoints between the two treatment groups will be calculated as well as their 95% confidence intervals. Student’s t tests, Chi square tests or Fisher exact tests will be applied where appropriate. The risk of re-operation will also be studied by application of a multiple logistic regression model.

### Statistics

*Sample size calculation*: This is a superiority design: Our hypothesis is that length of hospital stay is shorter after incisional hernia repair, and therefore the laparoscopic approach will be superior in terms of cost-effectiveness. Statistics are based on an average hospital stay of 2 days (SD 5) after laparoscopic repair [8]. The outcome is considered superior if there is a difference in hospital stay of more than 2 days. Therefore, 135 patients in each treatment arm are needed (alpha 0,05/power 0,9) [16]. Loss to follow-up may occur; we will therefore aim for an inclusion of 300 patients.

*Randomisation* will be performed through a computer-guided system. A stratified block-randomisation will be used per centre. The randomisation code will be noted on the patients file. Peritonitis carcinomatosa, unplanned surgical procedures for pathology that was not discovered during pre-operative analysis, absence of an incisional hernia are reasons for post-randomization exclusion.

### Feasibility

Twelve hospitals, both university medical centres and community hospitals, will participate in this trial. The study group consists of hernia experts from these 12 centres, who frequently perform laparoscopic as well as open hernia surgery. These hospitals perform about 20-30 incisional hernia corrections a year, and aim for an inclusion of 10-15 patients each year.

We aim for 125 patients per year, hence 12-13 inclusions every month. This is highly feasible, because these 12 centres together perform over 300 incisional hernia repairs annually. We aim for participation of more centres along the way, but only experienced laparoscopic surgeons can participate.

### Time schedule

Study preparation and formation of a core study group is already in progress. Initiation of the INCH-trial around 1-8-2012 after METC permission is obtained in each hospital. We aim for an inclusion rate of 10-15 patients per month. About 28 months are needed to include the amount of patients needed to calculate the difference in length of hospital stay. After this period the trial will continue; to meet the secondary end-point a longer follow-up period is needed. The follow-up will be continued at 3 and 5 year after index surgery. Patients who don’t want to participate and patient who are excluded will be registered.

### Ethical approval and safety monitoring

According to the ‘Good Clinical Practice’ rules, ethical approval has been asked and obtained from the Medical Ethical Board (METC) of the VU University Medical Center. This is an independent board and they will supervise the trail and make decisions about all possible changes in the study through amendments. The board will also monitor the possible complications.

No experimental surgery is performed; all the surgical techniques used are already part of our daily practice. All hospitals record (post-operative) morbidity according to guidelines of the Dutch Society of Surgeons. Adverse effects will be registered and told to the METC.

#### SAE’s

All individual Serious Adverse Events (SAE’s) will be registered and reported to the CCMO. Sepsis and possible re-operation due to a missed bowel perforation after laparoscopic incisional hernia repair, as well as death in the direct post-operative phase will be reported within 7 days. Permission has been obtained for ‘line listing’ for all other individual SAE’s; the METC permits to report all other events once every 6 months.

#### Criteria for participating centres

Participating surgeons have already performed at least 50 open and 50 laparoscopic incisional hernia repairs. They will hand over an unedited recording of a laparoscopic incisional hernia repair of their own. The principle investigators will review their recordings. Guidelines to perform a safe laparoscopic procedure will be made. Participating surgeons have to follow the guidelines.

### Goal

The goal of the trial is to establish whether laparoscopic incisional hernia repair is superior to conventional open incisional hernia repair in terms of cost-effectiveness. This is measured through length of hospital stay and quality of life. Secondary endpoints are re-operation-rate (due to complications or recurrence), morbidity, mortality and shape of the abdomen.

In current surgical practice there is on going discussion about the possible benefits of laparoscopic incisional hernia surgery. Scientific evidence is lacking to determine whether laparoscopic correction is superior to conventional open techniques in terms of cost-effectiveness.

## Discussion

The difference in time to full recovery between the two treatment strategies is thought to be in favor of laparoscopic incisional hernia repair. Laparoscopic incisional hernia repair is therefore expected to be a more cost-effective approach.

## Competing interests

The authors declare that they have no competing interests.

## Authors’ contributions

MMP, writer of this manuscript, chief investigator, corresponding autor. VU University Medical Center/Alkmaar Medical Center. JAA, chief investigator of one of the participating centers (Leeuwarden Medical Center). JGHB, chief investigator of one of the participating centers (Alkmaar Medical Center). HC, chief investigator of one of the participating centers (Red Cross Hospital, Beverwijk). EC, chief investigator of one of the participating centers (Meander Medical Center, Amersfoort). JDD, co-writer of the manuscript, will be the next chief inverstigator. BJD, chief investigator of one of the participating centers (Slotervaart Hospital, Amsterdam). NG, chief investigator of one of the participating centers (Tergooi Hospital, Hilversum). ESML, supervising statistic, epidemiologist. JFL, chief investigator of one of the participating centers (Erasmus University Medical Center, Rotterdam). RKJS, chief investigator of one of the participating centers (University Medical Center, Utrecht). MPS, chief investigator of one of the participating centers (OLVG Hospital, Amsterdam). DJAS, chief investigator of one of the participating centers (West Fries Gasthuis, Hoorn). WHS, chief supervisor of the INCH trial, Alkmaar Medical Center. HJB, chief supervisor of the INCH trial, VU University Medical Center Amsterdam. All authors read and approved the final manuscript.

## Pre-publication history

The pre-publication history for this paper can be accessed here:

http://www.biomedcentral.com/1471-2482/13/18/prepub

## References

[B1] Cassar K Munro A Surgical treatment of incisional hernia Br J Surg 2002 89 534 545 10.1046/j.1365-2168.2002.02083.x 11972542

[B2] Den Hartog D Dur AH Tuinebreijer WE Kreis RW Open surgical procedures for incisional hernias Cochrane Database Syst Rev 2008 3 CD006438 18646155 10.1002/14651858.CD006438.pub2 PMC8924951

[B3] Llaguna OH Avgerinos DV Lugo JZ Matatov T Abbadessa B Martz JE Leitman IM Incidence and risk factors for the development of incisional hernia following elective laparoscopic versus open colon resections Am J surgaug 2010 2 200 265 269 10.1016/j.amjsurg.2009.08.044 20122681

[B4] Rosch R Junge K Knops M Lynen P Klinge U Schumpelick V Analysis of collagen-interacting proteins in patients with incisional hernias Ach surg 2003 387 427 432 10.1007/s00423-002-0345-3 12607124

[B5] Nieuwenhuizen J Kleinrensink GJ Hop WC Jeekel J Lange JF Indications for incisional hernia repair: an international questionnaire among hernia surgeons Hernia 2008 12 223 225 10.1007/s10029-007-0322-4 18085346

[B6] Flum DR Horvath K Koepsell T Have outcomes of incisional hernia repair improved with time? Ann Surg 2003 237 1 129 135.10 10.1097/00000658-200301000-00018 12496540 PMC1513979

[B7] Forbes SS Eskicioglu C McLeod RS Okrainec A Meta-analysis of randomized controlled trials comparing open and laparoscopic ventral incisional hernia repair with mesh Br J Surg 2009 96 851 858 10.1002/bjs.6668 19591158

[B8] Sajid MS Bokhari SA Mallick AS Cheek S Baig MK Laparoscopic versus open repair of incisional/ventral hernia: a meta-anaysis Am J Surg 2009 197 64 72 10.1016/j.amjsurg.2007.12.051 18614144

[B9] Luijendijk RW Hop WCJ van den Tol MP de Lange DCD Braaksma MMJ IJzermans JNM Boelhouwer RU de Vries BC Salu MKM Wereldsma JCJ Bruijninckx DCMA Jeekel J A comparison of suture repair with mesh repair for incisional hernia N Engl J Med 2000 343 392 398 10.1056/NEJM200008103430603 10933738

[B10] Muysoms FE Miserez M Berrevoet F Campanelli G Champault GG Chelala E Dietz UA Eker HH El Nakadi I Classification of primary and incisional abdominal wall hernias Hernia 2009 13 407 414.11 10.1007/s10029-009-0518-x 19495920 PMC2719726

[B11] Brazier J The estimation of a preference-based measure of health from the SF-36 J Health Econ 2002 21 2 271 292 10.1016/S0167-6296(01)00130-8 11939242

[B12] Oostenbrink JB Oostenbrink JB Bouwmans CAM Koopmanschap MA Rutten FFH Handleiding voor kostenonderzoek: Methoden en standaard kostprijzen voor economische evaluaties in de gezondheidszorg. Geactualiseerdeversie 2004 [Handbook for cost studies: methods and standard costs for economic evaluation in health care. Updatedversion 2004] 2004 Den Haag, The Netherlands: College voor Zorgverzekeringen

[B13] van Buuren S Oudshoorn CGM Multivariate Imputation by Chained Equations 2000 Leiden: TNO

[B14] Thompson SG How should cost data in pragmatic randomised trials be analysed? BMJ 2000 320 7243 1197 1200 10.1136/bmj.320.7243.1197 10784550 PMC1127588

[B15] Fenwick E A guide to cost-effectiveness acceptability curves Br J Psychiatry 2005 187 106 108 10.1192/bjp.187.2.106 16055820

[B16] Dupont WD Plummer WD Power and sample size calculations for studies involving linear regression Control Clin Trials 1998 19 589 601 10.1016/S0197-2456(98)00037-3 9875838

